# Primary Angle Closure Glaucoma-associated Genetic Polymorphisms in Northeast Iran

**DOI:** 10.18502/jovr.v15i1.5942

**Published:** 2020-02-02

**Authors:** Ali Yousefian, Saeed Shokoohi-Rad, Mohammad Reza Abbaszadegan, Dorsa Morshedi Rad, Selma Zargari, Saman Milanizadeh, Negar Morovatdar, Ramin Daneshvar

**Affiliations:** ^1^Eye Research Center, Mashhad University of Medical Sciences, Mashhad, Iran; ^2^Medical Genetics Research Center, Mashhad University of Medical Sciences, Mashhad, Iran; ^3^Immunology Research Center, Avicenna Research Institute, Mashhad University of Medical Sciences, Mashhad, Iran; ^4^Clinical Research Unit, Faculty of Medicine, Mashhad University of Medical Sciences, Mashhad, Iran

**Keywords:** Polymorphism, Primary Angle Closure Glaucoma, rs3816415, rs736893, rs7494379, rs1258267

## Abstract

**Purpose:**

To evaluate the association of five different polymorphisms from a genome-wide-associated study with susceptibility to glaucoma in the northeast Iranian population.

**Methods:**

Hundred and thirty patients with primary angle closure glaucoma (PACG) and 130 healthy controls were genotyped for the polymorphic regions with the aid of tetra-amplification refractory mutation system-polymerase chain reaction. The association of these variants with the disease susceptibility was measured statistically with the logistic regression method.

**Results:**

Hundred and thirty patients with PACG (53 males, 77 females) with a mean age of 64.5 ± 6.2 years and 130 healthy control subjects (51 males, 79 females) with a mean age of 64.0 ± 5.7 years were selected for evaluation. There was a significant association between rs3816415 (*P* = 0.005), rs736893 (*P* < 0.001), rs7494379 (*P* < 0.001), and rs1258267 (*P* = 0.02) with PACG susceptibility. This association could not be shown for rs3739821.

**Conclusion:**

It was revealed that studied variants in *GLIS3, EPDR1, FERMT2, *and *CHAT *genes can contribute to the incidence of PACG. Additional studies in other populations are needed to evaluate *DPM2-FAM102A*.

##  INTRODUCTION

Glaucoma is one of the leading causes of blindness worldwide. Approximately 70 million people are affected and it is predicted that by 2020, this number will rise to around 79.6 million globally.^[1]^ The incidence of glaucoma varies in different populations and geographic regions. While primary open-angle glaucoma (POAG) is the most common type, primary angle closure glaucoma (PACG) is associated with a more severe presentation and bilateral blindness.^[2,3,4]^ The visual function in PACG could be saved if early and proper treatments are adopted.^[5]^

PACG is characterized by apposition between the peripheral iris and trabecular meshwork, which can ultimately lead to compromised outflow and high intraocular pressure (IOP).^[6]^ Stress induced by IOP on optic disc results in compression, deformation, and remodeling of the lamina cribrosa with subsequent mechanical axonal damage and disruption of axonal transport or ischemic damage to the neural tissue.^[7]^

PACG is a complex disease influenced by a combination of environmental and genetic risk factors. Recently, *COL18A1*, which encodes collagen type XVIII, has been identified as a gene that affects angle closure in humans and can lead to PACG.^[8]^ An unusually higher incidence among first-degree relatives of affected patients compared with the general population suggests that genetic risk factors may play an important role in the development of PACG.^[9,10]^ Another factor indicating a genetic influence in PACG development is the heritability for narrow-angle and a shallow anterior chamber (two important causes of disease) that are approximately 49% and 93%, respectively.^[9,11]^

Although several genome-wide association studies (GWASs) for PACG revealed multiple genetic variants correlated with disease susceptibility, these results demonstrate that the exact mechanisms by which the culprit gene could cause PACG is not completely understood and the association of different single nucleotide polymorphisms (SNPs) with glaucoma are still controversial.^[12,13]^

Among different reported variants, expression quantitative trait locus (eQTL) mapping databases indicate that rs7494379 on chromosome 14 position 53,411,391, gene locus *FERMT2*, rs736893 on chromosome 9 position 4,217,028, gene locus *GLIS3,* rs1258267 on chromosome 10 position 50,895,770, gene locus *CHAT*, rs3816415 on chromosome 7 position 37,988,311, gene locus *EPDR1,* and rs3739821 on chromosome 9 position 130,702,477, gene locus *DPM2-FAM102A* are significantly expressed in ocular anterior segment tissues such as iris, ciliary body, and trabecular meshwork,^[14,15]^ which mark them as potential variants for PACG incidence.

The current study aimed to evaluate the association of these five SNPs evaluated in GWAS with PACG susceptibility in the northeast of Iran.

##  METHODS

##  Population study

This case-control study was conducted on subjects that were following up for PACG at Khatam Eye Hospital, a referral eye center in Mashhad, northeast of Iran, between 2017 and 2018. The case group included 130 patients with PACG that were diagnosed and followed by a glaucoma-trained ophthalmologist according to optic nerve exam, gonioscopy, IOP, and visual field changes and completed the interview of competency (53 males and 77 females). Individuals with primary angle closure and primary angle closure suspect (with only obstructed angle and/or increased IOP with no obvious optic nerve cupping or visual field defect) were not selected for the study and only patients with established optic nerve, head damage, or glaucomatous perimetric changes due to PACG were considered for evaluation. Control subjects were 130 healthy individuals (51 males and 79 females)^[16]^ without any glaucoma findings or signs of angle closure in gonioscopy. Clinical information including age and sex was gathered. The Ethics Committee of the Mashhad University of Medical Sciences approved the study.

##  SNP selection criteria

The analyzed polymorphisms were selected on the basis of recent GWAS analysis and the databases prepared on the National Center for Biotechnology Information SNP Database (https://www.ncbi.nlm.nih.gov/snp/) (Access date: Dec 20, 2018) and literature searches. We selected five validated SNPs with a minor allele frequency >1%.^[17,18]^

##  Genotyping

DNA was extracted from the whole blood using the standard salting-out method which was previously described by Miller et al.^[19]^ Genotyping was carried out by tetra-amplification refractory mutation system-polymerase chain reaction (tetra-ARMS PCR). For amplification of the sequences containing polymorphic site, compatible and specified primers were designed using GeneRunner v3.01 (http://generunner.net/) and NCBI BLAST was used (https://www.ncbi.nlm.nih.gov/tools/primer-blast/) for ensuring the specificity. Properties of designed primers are listed in Table 1.

**Table 1:** Tetra-ARMS primer details.


**SNP**	**Sequence**	**Product length (nucleotide)**	**Tm (degree centigrade)**
**rs736893**	forward 5'TGCTACCAGGACTTGTGGTTGTG3'	240	57
	reverse 5'CTATGTTCTTCCCAGCACACATTC3'		
	forward 5'ACAATAGCCTAAGAGCACAGAGG3'	130		
	reverse 5'GGAACCATGACTCTTGGATTTAAA3'		
**rs1258267**	forward 5'GAGGAAGGCTCATTGCGATGG3'	327	61
	reverse 5'TCCTGACTCAAATCTCCTGCCTTC3'		
	forward 5'TGAGATTCTGATGAGCAAGTGCATG3'	130		
	reverse 5'CCAGGTTGCCTGCACCTGCT3'		
**rs3816415**	forward 5'TGGTGGCTTGGTCAATCTG3'	216	57
	reverse 5'TCATGTGCCTAGTGTTTATAAACA3'		
	forward 5'ATTACTAGCTAGGCAATCACTTTAC3'	96		
	reverse 5'ATGCTCGGTCTGACCTGTG3'		
**rs3739821**	forward 5'AGAAGATCGTTACCTGCCAGCC3'	215	61
	reverse 5'GGGAACACACTCACACCTCGTG3'		
	forward 5'CGAGTGTGCAGCCTGACCAGT3'	145		
	reverse 5'AGTGACTTGCCTGTCCCAGAGAG3'		
**rs7494379**	forward 5'GCACCATTCCACCAAATAAGCAC3'	286	60
	reverse 5'CTTAACGTGATCATTAAGTATGGTATTCA3'		
	forward 5'TCCACTTCTGTGAGATGCAATGTAC3'	155		
	reverse 5'CATTTATGTTGGAGTTGCATGTTAGG3'		
SNP, single nucleotide polymorphism			

**Table 2:** Characteristics of the study groups


**Study Groups**	**PACG patients**	**Control Group**	**** ***P*** **-value**
**Number of Subjects**	130	130	
**Gender**		0.450
	**Male, n (%)**	53(40.8)	51(39.2)	
	**Female, n (%)**	77(59.2)	79(60.8)	
**Age (years)**		
** Mean (SD) **	64.5(6.2)	64.0(5.7)	0.657
n, number; PACG, primary angle closure glaucoma; SD, standard deviation				

**Table 3:** Genotype frequencies


**Genotypes**	**Snps**	**PACG patients N (%)**	**Controls N (%)**	**OR (95%CI)**	**P-value**
**rs736893**			< 0.001
	G/G	10(7.6)	46(35.3)	Reference	Reference
	G/A	120(92.4)	84(64.7)	6.77(3.2–14.2)	< 0.001
	A/A	0	0	–	–
**rs1258267**			0.02
	A/A	97(74.6)	113(86.9)	Reference	Reference
	A/G	30(23)	17(13.1)	2(1–3.9)	0.033
	G/G	3(2.4)	0	1.5(1.3–1.7)	0.078
**rs3816415**			0.005
	C/C	120(92.3)	129(99.2)	Reference	Reference
	C/T	10(7.7)	1(0.8)	10.75(1.3-85.4)	0.025
	T/T	0	0	–	–
**rs3739821**			0.42
	T/T	28(21.5)	23(17.6)	Reference	Reference
	T/C	79(60.7)	76(58.4)	1.69(0.7–3.7)	0.18
	C/C	23(17.8)	31(24)	1.46(0.7–2.7)	0.23
**rs7494379**			< 0.001
	C/C	48(36.9)	80(61.5)	Reference	Reference
	C/T	50(38.4)	37(28.4)	2.28(1.3-3.9)	0.004
	T/T	32(24.7)	13(10.1)	4.08(1.9-8.5)	< 0.001

**Table 4:** Genotype frequencies in male and female groups


**Genotypes**	**Gender**	**PACG patients N (%)**	**Controls N (%)**	**OR (95%CI)**	**P-value**
**rs736893**			<0.001
**G/G**	Male	5(9)	19(37)	Reference	Reference
**G/A**	48(91)	32(63)	5(1.5–16.5)	0.007
**A/A**	0(0)	0(0)	–	–
**G/G**	Female	5(7)	27(34)	Reference	Reference
**G/A**	72(93)	52(66)	7(2.5–22.2)	0.01
**A/A**	0(0)	0(0)	–	–
**rs1258267**			0.02
**A/A**	Male	39(74)	43(84)	Reference	Reference
**A/G**	12(22)	8(16)	2.7(0.8–9.2)	0.1
**G/G**	2(4)	0(0)	10(0.1–11.1)	0.98
**A/A**	Female	58(75)	70(89)	Reference	Reference
**A/G**	18(23)	9(11)	1.5(0.6–4)	0.3
**G/G**	1(2)	0(0)	11(0.6–27.4)	0.8
**rs3816415**			0.005
**C/C**	Male	47(89)	51(11)	Reference	Reference
**C/T**	6(11)	0(0)	18(0.2–23.4)	0.98
**T/T**	0(0)	0(0)	–	–
**C/C**	Female	73(95)	78(99)	Reference	Reference
**C/T**	4(5)	1(1)	3.6(0.3–25.4)	0.2
**T/T**	0(0)	0(0)	–	–
**rs3739821**			0.42
**T/T**	Male	12(23)	5(10)	Reference	Reference
**T/C**	28(53)	31(61)	0.4(0.1–1.5)	0.19
**C/C**	13(24)	15(29)	0.3(0.1–1.3)	0.11
**T/T**	Female	16(21)	18(23)	Reference	Reference
**T/C**	15(66)	45(57)	1(0.4–2.6)	0.83
**C/C**	10(13)	16(20)	0.7(0.2–2.4)	0.66
**rs7494379**			< 0.001
**C/C**	Male	16(31)	32(63)	Reference	Reference
**C/T**	22(41)	14(27)	2.6(0.9–7.4)	0.063
**T/T**	15(75)	5(10)	8.4(2.2–32.1)	0.002
**C/C**	Female	32(42)	48(61)	Reference	Reference
**C/T**	28(36)	23(29)	1.6(0.7–3.5)	0.2
**T/T**	17(22)	7(10)	3(1–8.8)	0.04

PCR reaction was performed in a final volume of 15 μl consisting 100 ng genomic DNA, 8 μl of PCR master mix (Ampliqon A/S, Stenhuggervej 22, DK-5230 Odense M, Denmark), 0.5 μM of inner primers, and 0.25 μM of outer primers. Amplified products were visualized by 2% agarose gel electrophoresis. To confirm the results, 10% of samples were randomly re-genotyped by direct sequencing on ABI3130xl genetic analyzer (Thermo Fisher Scientific, USA) and the results were reproducible, with no discrepancy.

##  Statistical analysis

Chi-square test was used for determining the statistical significance of non-association between different variables. Logistic regression analysis was performed to estimate the association between polymorphism variants and the risk of PACG in different genders. The odds ratios (OR) and 95% confidence intervals (CI) were adjusted. Reported *P*-values were two-sided and the significance level was considered < 0.05. The SPSS, version 23.0 program (SPSS, Inc., Chicago, IL) was used for statistical analysis.

##  Results

This study included 130 patients with PACG (mean age: 64.5 ± 6.2 years) and 130 healthy control subjects (mean age: 64.0 ± 5.7 years). Age and sex of the participants were comparable between the two groups (*P* > 0.05). Detailed properties of the study subjects are demonstrated in Table 2.

The tetra-ARMS PCR method was successfully applied to genotype five different SNPs. The association of genotypes and allelic frequencies of five polymorphisms with PACG was determined. Genotype frequencies of all tested polymorphisms were in Hardy–Weinberg equilibrium. Genotype frequencies of analyzed SNPs are demonstrated in Table 3.

As evident in Table 3, a highly significant association and perhaps predisposing effect was found for G/A genotype of rs736893 in the *GLIS3* gene and T/T genotype of rs7494379 in the *FERMT2* gene (*P* < 0.001). Statistically meaningful association of rs1258267 in the *CHAT* gene and rs3816415 in the *EPDR1* gene with PACG was also confirmed, but rs3739821 in the *DPM2-FAM102A* genes did not have any significant association with PACG frequency in our study population.

The correlation of rs736893, rs1258267, and rs7494379 with the disease was stronger in males than in females, and rs3816415 had more association with the disease in females [Table 4].

Overall, a highly significant (*P* < 0.001) predisposing effect for the C/T genotype of rs3816415 in the *EPDR1* gene and G/A genotype of rs736893 in the *GLIS3* was observed [Table 3]. Genotype frequency analysis revealed that heterozygote genotype in rs736893, A/A genotype in rs1258267, C/C genotype in rs3816415, and heterozygote genotype in rs3739821 were more frequent. However, genotype distribution in rs7494379 was not consistent and C/C genotype and C/T genotype were more frequent in cases and controls, respectively.

##  DISCUSSION

The results of the present study reveal significant differences in the frequencies of genotypes of *EPDR1, GLIS3, CHAT, and FERMT2* polymorphisms between PACG patients and controls. The significantly higher frequencies of genotype G/A of *GLIS3* and C/T of *EPDR1* in patients with PACG than those in the controls indicated that these genotypes may be associated with a susceptibility to this disease.

Previous studies showed controversial results on the association of PACG with genetic variants investigated in the current study. Zhuang *et al* found that only SNPs rs3753841 in* COL11A1*, rs1258267 in *CHAT,* and rs736893 in *GLIS3* are associated with PACG in northern Chinese people; however, other studies demonstrated *EPDR1, GLIS3, CHAT, FERMT2, *and *DPM2-FAM102A* polymorphisms contribution to the disease susceptibility.^[12,18,20]^

*FERMT2* encodes a protein called pleckstrin-homology-domain-containing family C member 1 (PLEKHC1), a component of the extracellular matrix, and could thus have a role in cell adhesion; cell–cell adhesion has been proposed as an important process in the pathogenesis of PACG.^[21]^ *GLIS3* is a member of the GLI-similar subfamily of Krüppel-like zinc-finger proteins.^[22]^ Earlier studies have shown that mutations in *GLIS3* cause neonatal diabetes and congenital hypothyroidism.^[23]^ SNP markers mapping close to* GLIS3 *have been observed to be significantly associated with type 1 diabetes in Europeans (rs7020673),^[24]^ type 2 diabetes in East Asians (rs7041847),^[25]^ and fasting plasma glucose levels in a large meta-analysis of European collections (rs7034200);^[26]^ however, metabolic pathways through which zinc-finger activation could contribute to pathogenesis of PACG is not well understood.^[12]^

*CHAT* on chromosome 10 encodes choline acetyltransferase, an enzyme responsible for the synthesis of the neurotransmitter acetylcholine, which has a role in pupillary constriction. Anticholinergic medications can precipitate acute PACG through pupillary dilatation mechanisms and subsequent pupillary block. Therefore, it is plausible that natural genetic variation in a gene influencing acetylcholine metabolism could alter the risk for PACG.^[27]^

*EPDR1* encodes a glycosylated type II transmembrane protein known as ependymin-related 1. It potentially has a role in cell adhesion, and it has some similarity to protocadherins and ependymins.^[26]^ As mentioned earlier, disturbance in cell–cell adhesion could have some roles in the pathogenesis of PACG.^[12]^

SNP rs3739821 is located in an intergenic region between* DPM2 *and* FAM102A*, a gene yet to be fully characterized. Mutations in *DPM2* have been linked to congenital defects in glycosylation,^[28]^ leading to severe pathological neurological phenotypes. Although not much is known about *FAM102A*, except that its expression is sensitive to the addition of β-estradiol, the nearby* PIP5KL1* gene has been reported to be involved in cell proliferation^[29]^ and potentially in tumorigenesis. Expression analysis revealed that all three genes (*FAM102A, DPM2, and PIP5KL1*) were expressed in all eye tissues tested, thus providing biological support for their potential role in PACG development.^[12]^

The expression of all these genes in the cornea, lens, retina, choroid, and optic nerve head confirms the function of these genes in these tissues.^[12]^ The activity of these genes products in the ocular system can explain how different variations in these genes may contribute to the PACG.

In conclusion, characterizations of these variations suggest that they can contribute to PACG susceptibility; however, the penetrance of the alleles may be low. Being able to divide the population into risk categories would allow tailored screening, prognosis, and treatment programs according to the risk of each individual. Additional studies in other populations with more participants should be considered to evaluate the association of *DPM2-FAM102A* with the disease.

##  Financial Support and Sponsorship

The authors would like to thank the Vice Chancellor of Research, Mashhad University of Medical Sciences, Mashahd, Iran (Grant # 950495).

##  Conflicts of Interest

There are no conflicts of interest.
